# Dose Optimization of Tyrosine Kinase Inhibitors in Chronic Myeloid Leukemia: A New Therapeutic Challenge

**DOI:** 10.3390/jcm10030515

**Published:** 2021-02-01

**Authors:** Alessandra Iurlo, Daniele Cattaneo, Cristina Bucelli, Massimo Breccia

**Affiliations:** 1 Hematology Division, Foundation IRCCS Ca′ Granda Ospedale Maggiore Policlinico, 20122 Milan, Italy; cattaneo.daniele@alice.it (D.C.); cbucelli@libero.it (C.B.); 2 Hematology, Department of Translational and Precision Medicine, Sapienza University, Policlinico Umberto 1, 00161 Rome, Italy; breccia@bce.uniroma1.it

**Keywords:** chronic myeloid leukemia, tyrosine kinase inhibitor, imatinib, dasatinib, nilotinib, bosutinib, ponatinib, treatment de-escalation, treatment-free remission, prognosis

## Abstract

The chronic myeloid leukemia (CML) therapeutic landscape has dramatically changed with tyrosine kinase inhibitor (TKI) development, which allows a near-normal life expectancy. However, long-term TKI exposure has been associated with persistent adverse events (AEs) which negatively impact on quality of life (QoL) and have the potential to cause significant morbidity and mortality. In clinical practice, TKI dose reduction is usually considered to reduce AEs and improve QoL, but dose optimization could have also another aim, i.e., the achievement and maintenance of cytogenetic and molecular responses. While therapy cessation appeared as a safe option for about half of the patients achieving an optimal response, no systematic assessment of long-term TKI dose de-escalation has been made. The present review is focused on the most recent evidences for TKIs dose modifications in CML clinical studies and in the real-life setting. It will consider TKI dose modifications in newly diagnosed patients, dose reduction for AEs, or in deep molecular response, either as a prelude to treatment-free remission (TFR) or as continuous maintenance therapy in those patients not wishing to attempt TFR. In addition, it will focus on patients not achieving a molecular response deep enough to go to TFR, and for whom dose reduction could be an option to avoid AEs.

## 1. Introduction

Chronic myeloid leukemia (CML) is a myeloproliferative neoplasm with an annual incidence of 1–2 cases per 100,000 persons, accounting for approximately 15% of newly diagnosed cases of leukemia in adults [1]. The therapeutic landscape of this disease has dramatically changed with the development of small molecule tyrosine kinase inhibitors (TKIs) targeting *BCR-ABL1*. The 10-year survival rate in CML-chronic phase (CP) has improved from approximately 20% to 80–90% [2], allowing a near-normal life expectancy [3].

TKIs, therefore, represent a milestone in CML treatment, and their goal is to achieve a major molecular response (MMR; a ≥3 log reduction in *BCR-ABL1* level corresponding to a *BCR-ABL1* level of ≤0.1% on the International Scale [IS]) within 12 months from starting treatment, and eventually a deep molecular response (DMR; defined as *BCR-ABL1* level of <0.01% on the IS), as well as preventing progression to accelerated (CML-AP) or blast phase CML (CML-BP) [4].

While being very effective, these drugs also have a toxicity profile that is frequently mild to moderate, but sometimes even serious. The regulatory approved dose of each TKI is fixed and often is derived from phase I safety studies designed to determine the maximum tolerated dose (MTD), followed by phase II and III studies of efficacy. It has to be underlined that though the MTD usually also represents the maximally effective dose (MED), in the case of CML the MED is defined as the dose associated with both maximal inhibition of *BCR-ABL1* and safety, especially when given over a long period of time. Based on these considerations, it is easy to consider why the MTD and MED might differ for each TKI [5]. Consequently, the MTD should not be selected blindly as the recommended phase II dose for efficacy testing, as it needs to be evaluated using all available information collected during the early stages of drug development. Therefore, definition of the optimal dose may need to be deferred until randomized phase II trials are conducted.

Currently, five TKIs are approved for CML treatment: imatinib, the first TKI registered for this indication; the second-generation TKIs, nilotinib, dasatinib, and bosutinib and finally ponatinib, a third-generation TKI. Imatinib, nilotinib, dasatinib, and bosutinib are recommended for both first and second or later lines of treatments; ponatinib can instead be used only for second or subsequent lines, representing at the moment the only TKI that can be effectively used also in the case of T315I mutation.

Long-term exposure to TKIs has been associated with persistent adverse events (AEs) with an impact on quality of life (QoL). Moreover, with second-generation TKIs an increasing evidence for more serious AEs has been recorded (Table 1) which in some patients can cause a significant morbidity and mortality as for instance pleural effusion (PE) and pulmonary arterial hypertension with dasatinib [6], dyslipidemia, and arterial thrombotic events with nilotinib [7,8], diarrhea, and liver dysfunction with bosutinib [9], and hypertension, arterial occlusive events (AOEs), and pancreatic dysfunction with ponatinib [10].

Evidence is accruing that TKI dose modifications are safe and feasible throughout CML treatment and are an important consideration for the prevention and management of AEs, improving adherence and reducing treatment interruptions [11]. Furthermore, dose optimization together with the prevention of AEs might also pursue the aim of achieving and maintaining cytogenetic and molecular response, since trials of intermittent TKIs treatment have shown that responding patients are often overtreated [12].

It must be underlined that TKI dose optimization should be considered early as, once chronic toxicities developed, the beneficial effect of this approach is still a matter of debate, especially in a few specific settings [13]. While therapy cessation appears as a safe option for about half of the patients achieving an optimal response [14], so far, no systematic assessment of long-term TKI dose de-escalation has been made. By means of a mathematical model, Fassoni et al. firstly reported a strong evidence suggesting that for most patients who have already achieved a sustained remission, a TKI dose de-escalation of at least 50% does not lead to a reduction of long-term treatment efficacy and maintains a secondary decline of *BCR-ABL1* levels [15]. In detail, it can be assumed that the initial treatment phase is dominated by the cytotoxic TKI effect on proliferating leukemia stem cells (LSCs), which leads to a rapid reduction in *BCR-ABL1* levels, as suggested by imatinib dose-escalation studies indicating that a higher TKI dose leads to a more rapid response [16,17]. Similarly, after depletion of initially abundant proliferating LSCs, the treatment response is dependent upon the rare activation of quiescent LSCs, providing a consistent explanation for the slower long-term decrease in proliferating and quiescent LSCs [18]. In addition, it has been shown that a continuous *BCR-ABL1* monitoring can provide a patient-specific prediction of an optimal reduced dose without decreasing the anti-leukemic effect on residual LSCs, suggesting that dose-halving should be considered as a long-term treatment option for CML patients with good response under continuing maintenance TKI therapy with the clinical potential of reducing both treatment-related AEs and overall treatment costs.

The present review is focused on the most recent findings on TKIs dose modifications throughout the CML patients’ treatment journey in clinical studies (Table 2) as well as in the real-life setting (Table 3). It will deal with the issues of dose modification for each TKI in newly diagnosed patients including the elderly, dose reduction during therapy often but not only for AEs, and dose reduction for patients in DMR, either as a prelude to an attempt at treatment-free remission (TFR) or as continuous maintenance therapy in those patients not wishing to achieve a TFR.

## 2. Tyrosine Kinase Inhibitors

### 2.1. Imatinib

Imatinib (Glivec; Novartis), the first TKI, completely changed CML patients’ prognosis [48]. It was approved in 2001 in both the US and Europe for the treatment of all CML phases and, as its patent has expired, is now available as a generic drug [49].

The introduction of TKIs has dramatically improved patients’ survival so that CML is now considered as a chronic disease. Indeed, an update of the International Randomized Study of Interferon and STI571 (IRIS) trial showed that the estimated 10-year overall survival (OS) of imatinib-treated patients was 83.3% [19], and several studies based on population cancer registries have shown that CML five-year survival has increased since TKIs became available.

However, many issues remain unsolved, including the definition of the best TKI, dose and schedule for each CML patient, as well as the identification of the best strategy to reduce AEs frequency and severity [50]. Michel et al. by analyzing the data of the German CML-Study IV [20] tried to deal with two of these problems, i.e., the best TKI dose and AEs reduction [51]: in particular, they report that 90% of the subjects initially randomized to receive high-dose imatinib, i.e., 800 mg/day, and achieving a stable MMR, could reduce the dosage to 400 mg/day without losing their molecular response, with the benefits of preventing AEs and reducing costs, and likely increasing compliance.

Several previous clinical trials investigated whether high-dose imatinib was more effective than the approved dose of 400 mg/day [16,21,22], with the primary endpoint most frequently represented by the proportion of patients achieving an MMR at one year [52]. While imatinib at 800 mg/day usually led to a faster MMR, a similar response rate was however recorded at one or two years; moreover high-dose imatinib was associated with an increased rate of grade ≥3 AEs and a worse compliance [16,21,22]. As a consequence, many patients originally assigned to high-dose imatinib were shifted to 400 mg/day. A subsequent landmark analysis of the German CML-Study IV reported that subjects receiving an optimized high-dose of imatinib (median dose 600 mg/day) achieved deeper and faster molecular responses compared with those treated with 400 mg/day, with no increase in grade ≥3 AEs [23]. Furthermore, the conventional and optimized strategies of giving imatinib resulted in similar event-free survival, progression-free survival (PFS), and OS.

The report of Michel et al. recalls another interesting observation previously made in the Italian INTERIM study. In this trial, older (>65 years) CML subjects treated with imatinib 400 mg/day and stopping it every three months were able not only to maintain an MMR in the majority of cases but sometimes even to improve the depth of the molecular response [12]. At a minimum follow-up of 6 years, 21% of patients have lost complete cytogenetic response (CCyR) and MMR, whereas 21% MMR only. Importantly, when imatinib was reassumed at the same dose, all the patients achieved again CCyR and MMR or even DMR, with a probability of remaining on the study schedule of 48% at six years [53]. An obvious explanation of the different MMR rate reported in the two studies is that these two studies substantially differ in the fact that, in the INTERIM trial, subjects completely stopped imatinib while in the German CML-Study IV patients only had an imatinib dose reduction.

Specifically concerning the topic of very elderly (>75 years) CML patients, they are sometimes treated at diagnosis with different imatinib dosages, based on concomitant diseases and physicians’ judgement. As the treatment of this patients’ subset might be improperly influenced by physicians’ personal perception, baseline evaluation of comorbidities according to specific tools, like the Charlson Comorbidity Index (CCI), can improve the initial decision-making [35,54]. As demonstrated by long-term follow-up data, imatinib can play a crucial role in the front-line treatment of very elderly CML patients without increased toxicity, and any effort to treat these subjects with standard doses should, therefore, be made in order to achieve the same responses as in younger cases [36,37,38,39].

### 2.2. Dasatinib

The superiority of dasatinib in inducing faster and deeper responses at early time-points in comparison with imatinib was firstly reported in the DASISION trial; the five-year follow-up have however clearly shown that drug-related AEs, i.e., PE, occurred more frequently in the dasatinib cohort (28% vs. <1%), thus representing the leading cause of its discontinuation [24]. Additional AEs caused by dasatinib also include myelosuppression (20%) and, occasionally, pulmonary arterial hypertension (5%) [55,56].

In a retrospective analysis of the DASISION trial, dasatinib dose reductions did not affect efficacy, and the superior MMR rate was maintained while improving its safety profile [56]. In addition, the seven-year follow-up of the CA180-034 study, which involved both imatinib-resistant and -intolerant patients switched to dasatinib, has demonstrated that 100 mg once daily dose was as effective as both 140 mg once daily or 70 mg twice daily but with a more favorable toxicity profile [25].

More recently, a lower dose (50 mg once daily) was evaluated in patients with newly diagnosed CML and proved to be effective and safe as initial therapy for CML-CP. High response rates and rapid achievement of CCyR were observed in nearly all patients after six months from the start of therapy and 12-month MMR and DMR rates were 79% and 46%, respectively. Furthermore, these results were achieved with a very favorable toxicity profile [2]. At a longer follow-up, the efficacy and toxicity profile of lower dose, in comparison with historical data from the DASISION trial, were more favorable, in terms of both drug exposure improvement and minimization of drug interruptions due to AEs [57].

The relevance of dasatinib dosage was highlighted also in a real-life cohort of CML patients older than 65 years treated frontline: the starting dose was 100 mg/day in 54 patients (83.0%), whereas 11 patients (17.0%) received less than 100 mg/day. Pleural effusions of all WHO grades occurred in 12 patients (18.5%) and all 10 subjects who needed a permanent treatment discontinuation because of toxicity were treated with 100 mg/day as an initial dose [40].

In another retrospective analysis involving 21 CML-CP patients aged ≥65 years, low-dose dasatinib generated an adequate molecular response without causing severe AEs. In details, 72% of the patients received a mean dasatinib dose of ≤20 mg/day and achieved an MMR in 94% and a DMR in 74% of the cases [41].

An additional study who retrospectively collected data from a real-life series of 853 dasatinib-treated patients in both first and later lines of therapy, have identified 196 cases of PE (incidence 23.0%). The majority of these cases were treated with dasatinib 100 mg/day (70.4%), less than 100 mg/day in 14.3%, and more than 100 mg/day in the remaining subjects; unfortunately, dose reduction after the first episode of PE was not able to prevent its recurrence. Interestingly, since at first PE development 28.6% of patients were already in MMR and 37.8% in DMR, it might be speculated that a dose reduction before the PE occurrence in patients who have already achieved an MMR or a DMR could help to reduce the rate of this specific AE [42]. As an alternative option, an “on/off” treatment schedule with a weekend drug holiday could also be considered [26].

### 2.3. Nilotinib

The different efficacy and safety profile of nilotinib according to its starting dose has been already reported in the ENESTnd study demonstrating the equivalent efficacy of nilotinib 300 mg and 400 mg twice daily (BID) [27], even if only nilotinib 400 mg BID showed superior five-year OS compared to imatinib (imatinib 91.7%, nilotinib 300 mg BID 93.7%, and 400 mg BID 96.2%; *p* = 0.0266). However, this finding was countered by a higher rate of cardiovascular events in the nilotinib 400 mg BID arm (cardiovascular events of any grade: imatinib, 2.1%; nilotinib 300 mg BID, 7.5%; and 400 mg BID, 13.4%). Consequently, the recommended starting dose of nilotinib for newly diagnosed patients is 300 mg BID and 400 mg BID only for second or later lines of therapy [58].

Interestingly, in a retrospective analysis of efficacy and safety of frontline nilotinib in 55 older CML patients regularly followed in 18 Italian hematological centers, the presence of comorbidities in a higher proportion of cases did not affect the molecular response rate, as well as the tolerability of the drug. However, as eight patients definitively discontinued nilotinib because of AEs and 11 continued the drug at a lower dose, in the long-term treatment it might be advisable to adapt the dosing schedule in order to improve the compliance, while maintaining an optimal molecular response [43].

In the NILO-RED study after achievement of MMR on standard nilotinib schedule, patients were switched to a low-dose regimen as maintenance therapy; subjects on both first-line (*n* = 46, 300 mg BID) and second-line nilotinib (*n* = 21, 400 mg BID) were involved. After nilotinib dose reduction, two of the 46 first-line patients and none of the second-line subjects lost MMR which however was spontaneously regained 4 and 6 months later. All patients reducing dosages in DMR and eight out of 10 patients reducing it in MMR maintained response at 12 months. In addition, nine patients in long-lasting DMR stopped nilotinib remaining treatment-free at a median follow-up of 18 months. The NILO-RED study provides therefore a preliminary evidence that a switch to nilotinib maintenance at a once daily dose is feasible and safe, regardless of prior therapies [28].

In the second-line setting, the ENESTswift trial supported a strategy of crossing to nilotinib 300 mg BID in patients intolerant to first-line imatinib or dasatinib. Accordingly, this approach appears effective and well-tolerated in most CML-CP patients [29].

### 2.4. Bosutinib

The original first-line study of bosutinib in newly diagnosed CML-CP patients failed its primary endpoint of demonstrating a cytogenetic response superior to imatinib, partly due to the AEs profile at the recommended dose of 500 mg once daily [9]. The subsequent BFORE trial, using in the same clinical setting a lower bosutinib dose (400 mg once daily), has indeed highlighted its improved efficacy compared to imatinib, with a better tolerability profile than the 500 mg daily dose [30].

To further confirm this issue, the GIMEMA BEST study evaluated second-line bosutinib in elderly (≥60 years) patients resistant or intolerant to imatinib. In responsive subjects, the dosage was maintained (300 mg or 400 mg daily), the primary endpoint being the proportion of patients in MMR at one year. In the final results, bosutinib induced or maintained an MMR in 65% of patients (molecular improvement in 67%), being most subjects (79%) still on bosutinib, with 88% of them assuming 300 mg daily or less. Intolerance was the most frequent reason of permanent discontinuation, while no progression was observed. These results showed that in elderly patients second-line bosutinib may be highly effective and better tolerated at a daily dose lower than 500 mg, namely at 300 mg/day [31].

In addition, in a real-life cohort of 91 elderly CML patients bosutinib starting dose was 500 mg in only 22% of the cases, while in the large majority it was administered at less than 400 mg/day (300 mg/day in 28 patients [30.8%], 200 mg/day in 34 patients [37.3%] and 100 mg/day in two patients [2.2%]). Nevertheless, bosutinib was shown to be effective with a favorable safety profile also in elderly subjects with important comorbidities and resistant/intolerant to previous TKIs [44].

Generally considered safe from the cardiovascular point of view, bosutinib has been seldom associated with PE [59]; it could, therefore, play a significant role in the current clinical practice for this frail patient population.

### 2.5. Ponatinib

The proven efficacy of ponatinib in CML patients resistant to previous TKIs was demonstrated in the pivotal phase II PACE trial, which enrolled 449 patients including 270 CML-CP cases [10] either resistant or intolerant to dasatinib or nilotinib or with a T315I mutation; a five-year PFS and OS of 53% and 73%, respectively, were achieved. Nevertheless, some concerns emerged about the ponatinib-related cardiovascular (CV) safety profile when used at the recommended starting dose of 45 mg/day, since a clear relationship between CV AEs and dosage was observed. A post hoc analysis was then performed to assess the effect of prospective ponatinib dose reductions on maintenance of response. Among 145 CML-CP patients remained on ponatinib, dose reductions were implemented in 86 cases according to prospective recommendations; most of the remaining 59 CML patients (88%) were already receiving a reduced dose. Rates of major cytogenetic response (MCyR) and MMR maintenance were high, regardless of preemptive dose reduction. Currently there is the indication to reduce the daily dose in patients who have already achieved at least a MCyR [60,61].

The subsequent phase 2 OPTIC (NCT02467270) trial was designed to prospectively evaluate response-based ponatinib dosing regimens with the aim of optimizing its efficacy and safety in CML-CP patients. Ponatinib was evaluated at three starting doses (45 mg, 30 mg, or 15 mg daily) in subjects resistant/intolerant to ≥2 TKIs or with a T315I mutation, with the primary endpoint represented by *BCR-ABL1* levels at 12 months ≤1% on the IS. On achievement of this endpoint, or for safety reasons, doses were reduced to 15 mg in the 45-mg and 30-mg cohorts. At a median follow-up of about 21 months, the maximum benefit:risk ratio, regardless of mutational status or number of prior TKIs, was observed in patients treated with a 45-mg starting dose, with a reduction to 15 mg upon achievement of response. Furthermore, patients with the T315I mutation who initiated ponatinib at 45 mg experienced better response rates than those who began treatment at 30 mg or 15 mg. With regards to safety profile, there was a trend toward higher AEs rates in the 45-mg cohort, as well as for patients treated with ≥3 TKIs; rates of confirmed AOEs were however low (≤6%) in all three cohorts irrespective of the number of prior TKIs [32,33].

The OITI trial, a non-interventional study including adult patients with CML-CP, -AP or -BP across 40 Italian centers (academic and hospital settings) as of October 2018, showed a ponatinib favorable efficacy and safety profile in CML subjects treated according to routine clinical practice. By six months, most patients had achieved a CCyR and 44% of them obtained an MMR in second or later lines; furthermore, the probability of survival at two years was more than 90%. No new safety concerns emerged than those previously reported. Consequently, the early use of ponatinib (84% of patients received it as second or third line of treatment) as well as a careful dose selection appear crucial for the safety and efficacy outcomes observed [34].

In a retrospective real-life analysis of ponatinib in CML-CP subjects resistant and/or intolerant to prior TKIs, 15 mg/day as starting dose (off-label) or de-escalated dose (i.e., starting dose of 45 or 30 mg/day), turned out to be effective with an acceptable toxicity profile, suggesting a potential benefit also in these subsets of patients [62]. In addition, considering the high antileukemic potency expressed by ponatinib, it is conceivable that lower-dose regimens or full-dose induction followed by dose reduction might also prove to be useful for intolerant patients [45,46,47,63,64].

## 3. Dose Reduction before TFR

The majority of TFR studies in CML patients have discontinued TKI treatment abruptly and have focused only on patients with stable DMR.

The DESTINY study is a non-randomized, phase II trial undertaken at 20 UK hospitals, aiming to examine the effects of gradual treatment withdrawal before TKI discontinuation and whether TFR is feasible for patients with less deep but stable remission. In details, TKI treatment was de-escalated to half the standard dose for 12 months, then stopped for a further 24 months, with frequent PCR monitoring. The primary endpoint was the proportion of patients who could first de-escalate their treatment for 12 months, and then stop treatment completely for a further two years, without losing MMR. Three DMR and nine MMR patients experienced molecular recurrence during the de-escalation phase. In the DMR group, 67% of patients reached the 36-month trial completion point and recurrence-free survival was 72%. On the contrary, 33% of MMR entrants completed the study and recurrence-free survival was 36%. Importantly, all recurrences regained MMR within five months of treatment resumption, thus supporting the assumption that initial de-escalation before discontinuation might improve the success of TFR protocols [65].

When Fassoni et al., by means of their mathematical model, simulated the DESTINY discontinuation protocol for the IRIS and German CML-Study IV patients, they noticed a higher rate of molecular relapse in comparison to a structurally identical control group receiving full-dose treatment before TKI stop. However, analyzing dose reductions of different duration, they predicted a beneficial effect if patients remain at half-dose for longer before TKI discontinuation according to the molecular response just before de-escalation, thereby emphasizing that the full benefit of TKI dose de-escalation appears in the long term [15].

In a more recent retrospective analysis of 77 CML patients who discontinued TKIs therapy, 26 subjects have been managed with low-dose TKIs before stopping treatment. At a median follow-up of 61.5 months, TFR at 12 months was 56.8% in the full-dose TKI group and 80.8% in the low-dose group, while at 60 months it was 47.5% and 58.8%, respectively. The median time to MMR lost in the entire cohort was 6.2 months. Interestingly, all patients quickly achieved MMR after resuming TKI therapy, independently from both dose reduction and potential interferon-α pretreatment [66].

The ongoing DANTE trial is a prospective, single arm, phase II study aimed to assess the effect of nilotinib reduced to half the standard dose during a consolidation period of 12 months on TFR in CML-CP patients treated with first-line nilotinib who reached a sustained DMR before study entry. The primary endpoint is the percentage of patients in full TFR 96 weeks after the start of the consolidation phase. During the TFR phase, loss of MMR will cause re-initiation of nilotinib treatment at 300 mg BID [67].

Preliminary data concerning TFR feasibility after TKI de-escalation can be derived also from real-life experiences: in details, in an Italian retrospective study on low-dose ponatinib in 52 CML-CP patients intolerant to previous TKIs, one out of 15 patients who obtained a DMR with this treatment successfully achieved also a TFR [47].

## 4. General Considerations

Dose modifications may be considered at all stages of the patient’s treatment journey; at diagnosis could be applied for frail subjects as well as for elderly or those with multiple comorbidities and during follow-up for other categories who experience toxicity or in order to prevent it. The two main goals of this approach are to maintain the proved efficacy of TKIs while reducing side effects. Adverse events may be persistent and of low grade, such as the chronic fatigue and fluid retention associated with imatinib, mild diarrhea for bosutinib or more serious, such as PE associated with dasatinib, or life threatening as increased risk of AOEs seen with nilotinib and ponatinib. Dose modifications also improve adherence and reduce treatment interruptions, with consequent positive impact on clinical outcomes, QoL and healthcare costs. In addition, dose reduction could allow a wider use even of those TKIs not properly indicated in presence of comorbidities.

Moreover, dose reduction is also being considered in clinical trials prior to TFR attempts. For patients who are unable to stop therapy, treatment dose adjustment without jeopardizing the clinical outcome has important clinical implications. Combination of reduced TKIs dosage with newer (ABL001) or older approaches (immunomodulation) might represent a future answer to this issue.

Novel, prospective clinical trials are required to explore dose optimization in CML-CP, from newly diagnosed patients through those experiencing AEs, as a new promising treatment strategy.

## Figures and Tables

**Table 1:** Adverse events during TKI therapy.

	Imatinib	Dasatinib	Nilotinib	Bosutinib	Ponatinib
**Fluid retention**	**+++**	−	−	+	−
**Muscle cramps**	**+++**	−	−	−	−
**Fatigue**	++	++	++	++	++
**Rash**	++	**−**	**+++**	+/−	**+++**
**Nausea**	++	+/−	+	**+++**	++
**Diarrhea**	++	−	−	**+++**	−
**Increased pancreatic enzymes**	−	−	++	+	**+++**
**Hypertension**	−	++	++	−	**+++**
**Pleural effusion**	−	**+++**	−	+	−
**Arterial occlusive events**	−	−	++	−	**+++**

TKI: tyrosine kinase inhibitors; Bold values: most frequent adverse events for each TKI.

**Table 2:** Clinical trials evaluating different TKIs doses.

	Study	Daily Dose	Reference
**Imatinib**	IRIS	400 mg	[19]
	German CML-Study IV	800 mg	[20]
	Baccarani et al.	800 mg	[21]
	Cortes et al.	800 mg	[16]
	Cortes et al.	800 mg	[22]
	German CML-Study IV	600 mg	[23]
	INTERIM	400 mg STOP every three months	[12]
**Dasatinib**	DASISION	100 mg	[24]
	CA180-034	100 mg	[25]
	Naqvi et al.	50 mg	[2]
	La Rosée et al.	100 mg 3–5 days per week	[26]
**Nilotinib**	ENESTnd	300–400 mg BID	[27]
	NILO-RED	From BID to once daily	[28]
	ENESTswift	300 mg BID	[29]
**Bosutinib**	BELA	500 mg	[9]
	BFORE	400 mg	[30]
	BEST	400 mg vs. 300 mg	[31]
**Ponatinib**	PACE	45 mg	[10]
	OPTIC	45 mg vs. 30 mg vs. 15 mg	[32,33]
	OITI	45 mg vs. 30 mg vs. 15 mg	[34]

Abbreviations: BID, twice daily.

**Table 3:** Real-life studies evaluating different TKIs dosages.

	Study	Patients	Daily Dose	Reference
**Imatinib**	Breccia et al.	181	400 mg vs. 200–300 mg	[35]
	Latagliata et al.	211	400 mg vs. >400 mg vs. <400 mg	[36]
	Crugnola et al.	263	400 mg	[37]
	Cervantes et al.	43	400 mg vs. 300 mg	[38]
	Park et al.	9	400 mg vs. 200–300 mg	[39]
**Dasatinib**	Latagliata et al.	65	100 mg vs. <100 mg	[40]
	Itamura et al.	21	≤50 mg vs. ≤20 mg	[41]
	Iurlo et al.	196	100 mg vs. >100 mg vs. <100 mg	[42]
**Nilotinib**	Luciano et al.	55	300 mg BID	[43]
**Bosutinib**	Latagliata et al.	91	500 mg vs. <400 mg	[44]
**Ponatinib**	Iurlo et al.	7	15 mg	[45]
	Breccia et al.	29	45 mg vs. 30 mg vs. 15 mg	[46]
	Iurlo et al.	52	30 mg vs. 15 mg	[47]

Abbreviations: BID, twice daily.

## Data Availability

Not applicable.
